# Supplementary material for: Buca della Iena and Grotta del Capriolo: New chronological, lithic, and faunal analyses of two late Mousterian sites in Central Italy
Source: PLoS One. 2025 Jun 11;20(6):e0315876. doi: 10.1371/journal.pone.0315876 (PMC12157248; doi:10.1371/journal.pone.0315876)
Supplement: S1 File — (DOCX) [file pone.0315876.s001.docx]

OxCal code for the Buca della lena model:

Plot()

{

Outlier_Model("General",T(5),U(0,4),"t");

Sequence()

{

Boundary("Start 15");

Phase("15")

{

R_F14C("R00256.2/VERA-8487",0.00677,0.00061)

{

Outlier("General",0.05);

};

Combine("OxA-X-3211-32")

{

After()

{

Date(calBP(100000));

};

Before()

{

Date(calBP(47600));

};

};

};

Boundary("End 15/Start flowstone");

Phase("Flowstone")

{

};

Boundary("End flowstone/Start 14");

Phase("14 Start human occupation")

{

R_F14C("R00257.1/UCIAMS-286280",0.0051,0.0007)

{

Outlier("General",0.05);

};

};

Boundary("Transition 14/9");

Phase("9")

{

R_F14C("R00259.2/VERA-8488",0.00531,0.00061)

{

Outlier("General",0.05);

};

};

Boundary("Transition 9/6");

Phase("6")

{

R_F14C("R00255.1/UCIAMS-286278",0.0037,0.0007)

{

Outlier("General",0.05);

};

};

Boundary("Transition 6/5");

Phase("5")

{

R_F14C("R00253.1/UCIAMS-286276",0.0098,0.0007)

{

Outlier("General",0.05);

};

R_F14C("R00254.1/UCIAMS-286277",0.0086,0.0007)

{

Outlier("General",0.05);

};

};

Boundary("End 5");

};

};

| Name | Unmodelled (BP) | |  |  | Modelled (BP) | |  |  | Indices  Amodel 15.7  Aoverall 19 | | |  |  |
| --- | --- | --- | --- | --- | --- | --- | --- | --- | --- | --- | --- | --- | --- |
|  | from_68.3% | to_68.3% | from_95.4% | to_95.4% | from_68.3% | to_68.3% | from_95.4% | to_95.4% | Acomb | A | L | P | C |
| Boundary End 5 |  |  |  |  | 42260 | 41210 | 42500 | 39520 |  |  |  |  | 96 |
| R_F14C R00254.1/UCIAMS-286277 | 42640 | 42080 | 43040 | 41710 | 42530 | 42030 | 42910 | 41720 |  | 107.4 |  | 98.8 | 99.8 |
| R_F14C R00253.1/UCIAMS-286276 | 42190 | 41510 | 42410 | 41140 | 42330 | 41780 | 42520 | 41320 |  | 103.9 |  | 98.1 | 99.2 |
| Phase 5 |  |  |  |  |  |  |  |  |  |  |  |  |  |
| Boundary Transition 6/5 |  |  |  |  | 43530 | 42030 | 45130 | 41890 |  |  |  |  | 99.2 |
| R_F14C R00255.1/UCIAMS-286278 | 49270 | 45770 | 52380 | 44750 | 45530 | 42880 | 45920 | 42300 |  | 20.9 |  | 52.4 | 99.5 |
| Phase 6 |  |  |  |  |  |  |  |  |  |  |  |  |  |
| Boundary Transition 9/6 |  |  |  |  | 45810 | 44000 | 46400 | 42730 |  |  |  |  | 99.6 |
| R_F14C R00259.2/VERA-8488 | 45700 | 44190 | 46640 | 43220 | 45840 | 44440 | 46660 | 43520 |  | 103.6 |  | 98 | 99.7 |
| Phase 9 |  |  |  |  |  |  |  |  |  |  |  |  |  |
| Boundary Transition 14/9 |  |  |  |  | 46170 | 44670 | 47230 | 43970 |  |  |  |  | 99.7 |
| R_F14C R00257.1/UCIAMS-286280 | 46100 | 44250 | 47560 | 43250 | 46650 | 45060 | 47660 | 44450 |  | 94 |  | 97.4 | 99.7 |
| Phase 14 Start human occupation |  |  |  |  |  |  |  |  |  |  |  |  |  |
| Boundary End flowstone/Start 14 |  |  |  |  | 47450 | 45410 | 48970 | 44590 |  |  |  |  | 99.2 |
| Phase Flowstone |  |  |  |  |  |  |  |  |  |  |  |  |  |
| Boundary End 15/Start flowstone |  |  |  |  | 49100 | 46680 | 51620 | 45300 |  |  |  |  | 97 |
| -45649.5 |  |  |  |  | 47610 | 47590 | 47610 | 47590 |  |  |  |  | 100 |
| Before | ... | -45649.5 | ... | -45649.5 | -98049.5 | -45649.5 | -98049.5 | -45649.5 |  | 100 |  |  |  |
| -98049.5 |  |  |  |  | 100010 | 99990 | 100010 | 99990 |  |  |  |  | 100 |
| After | -98049.5 | ... | -98049.5 | ... | -98049.5 | -45649.5 | -98049.5 | -45649.5 |  | 100 |  |  |  |
| Combine OxA-X-3211-32 | 100000 | 47600 | 100000 | 47600 | 49360 | 47590 | 52720 | 47590 | 100 |  |  |  | 95 |
| R_F14C R00256.2/VERA-8487 | 43970 | 42860 | 44500 | 42560 | 49730 | 47300 | 53020 | 46010 |  | 5.4 |  | 0.8 | 95.7 |
| Phase 15 |  |  |  |  |  |  |  |  |  |  |  |  |  |
| Boundary Start 15 |  |  |  |  | 50800 | 47670 | 54670 | 47600 |  |  |  |  | 64.9 |
| Sequence |  |  |  |  |  |  |  |  |  |  |  |  |  |

**Table 1. Results of the Bayesian model generated for Buca della lena**. The data is rounded to the nearest 10 years.
